# DSMC Simulation and Experimental Validation of Shock Interaction in Hypersonic Low Density Flow

**DOI:** 10.1155/2014/732765

**Published:** 2014-01-30

**Authors:** Hong Xiao, Yuhe Shang, Di Wu

**Affiliations:** School of Power and Energy, Northwestern Polytechnical University, Xi'an, Shaanxi 710072, China

## Abstract

Direct simulation Monte Carlo (DSMC) of shock interaction in hypersonic low density flow is developed. Three collision molecular models, including hard sphere (HS), variable hard sphere (VHS), and variable soft sphere (VSS), are employed in the DSMC study. The simulations of double-cone and Edney's type IV hypersonic shock interactions in low density flow are performed. Comparisons between DSMC and experimental data are conducted. Investigation of the double-cone hypersonic flow shows that three collision molecular models can predict the trend of pressure coefficient and the Stanton number. HS model shows the best agreement between DSMC simulation and experiment among three collision molecular models. Also, it shows that the agreement between DSMC and experiment is generally good for HS and VHS models in Edney's type IV shock interaction. However, it fails in the VSS model. Both double-cone and Edney's type IV shock interaction simulations show that the DSMC errors depend on the Knudsen number and the models employed for intermolecular interaction. With the increase in the Knudsen number, the DSMC error is decreased. The error is the smallest in HS compared with those in the VHS and VSS models. When the Knudsen number is in the level of 10^−4^, the DSMC errors, for pressure coefficient, the Stanton number, and the scale of interaction region, are controlled within 10%.

## 1. Introduction

Shock interactions play an important role in hypersonic flows. In the past decade, significant efforts in computational fluid dynamics (CFD) have been exerted to develop prediction techniques for simulating these complex flow structures [1–3]. Most of these works were simulated using the continuum transport equations (Navier-Stokes), which describe the transport of mass, momentum, and energy. These equations are based on the hypothesis that the mean free-path length *λ* of gas molecules is very small compared with the characteristic dimension *L* of the flow. This characteristic dimension can be defined by the flowing gradient length scale

(1) $$\begin{array}{c} L=\frac{\phi }{\left(\partial \phi /\partial x\right)}. \end{array}$$

Here, *ϕ* is the flow property and it can be density or pressure and so on. Also, the Knudsen number Kn is usually used to describe low density flow:

(2) $$\begin{array}{c} \text{Kn}=\frac{\lambda }{L}. \end{array}$$

Flows with Kn > 10 are called “free molecular flows.” In this regime, intermolecular collisions rarely occur, and the flow is completely dominated by the interaction between the gas and the wall. Flows in the free molecular regime can be simulated using molecular dynamics (MD) or ballistic models. In the intermediate (0.01 < Kn < 10) or rarefied regime, both collisions, with solid surfaces and with gas molecules, are important and, therefore, have to be included in the simulation to obtain an accurate result. The direct simulation Monte Carlo (DSMC) method developed by Bird [4] is the perfect practical engineering method that can be used in the rarefied regime. It is also valid in the free-molecular and continuum regimes, although computational expenses become very large in the latter case. Its computational expenses, in fact, scale with the Knudsen number and become prohibitively large when Kn becomes lower than *∼*0.05. Usually, gas flow can be simulated for Kn < 0.01 using continuum-based CFD models, for Kn > 0.05 using the particle-based DSMC methods, and for 0.05 < Kn < 0.01 using the coupled NS/DSMC methods.

Unfortunately, hypersonic shock is not an entirely understood domain. Flow property *ϕ* changes significantly in the inner of shock. Also, the gradient of flow property ∂*ϕ*/∂*x* is large, because characteristic dimension *L* is small. It causes the Knudsen number becomes big in the inner of shock. And, the Knudsen number is also big in the far field which results in the simulation difficulty of low density hypersonic flows.

In low-density hypersonic flows, both CFD and DSMC are used in the prediction of pressure and heat characteristics. However, conclusions, drawn from the simulation and experiment, are different. Unfortunately, many CFD and DSMC simulations are incompatible with precision direction [5, 6].

Some double-cone flow simulation studies, including *Candler's ect* CFD [7] and Moss DSMC simulation [8], have been conducted. In these studies, comparisons between simulation and experiment reveal significant numerical problems that can be encountered when predicting strong gradients. Experimental data are used for comparisons, as conducted in ONERA R5Ch Mach 10 low-density wind tunnel [8]. In Moss's DSMC research, VHS model was used. HS and VSS models were not included in Moss's DSMC simulation. Both in *Candler's ect* CFD [7] and in Moss's DSMC [8] research, the experimental conditions of Run numbers 26, 32, 33, 36, and 38 are not included, as shown in Table 1.

For studying the influence of the DSMC molecular model on hypersonic shock interaction, DSMC simulations are conducted by three molecular models, namely, HS [9, 10], VHS [9, 10], and VSS [11]. The simulations are focused on experimental conditions of Run numbers 26, 32, 33, 36, and 38. These conditions are not included in previous studies. For further reliability, Edney's type IV shock interaction is also studied.

## 2. DSMC Model

**Figure 1:**
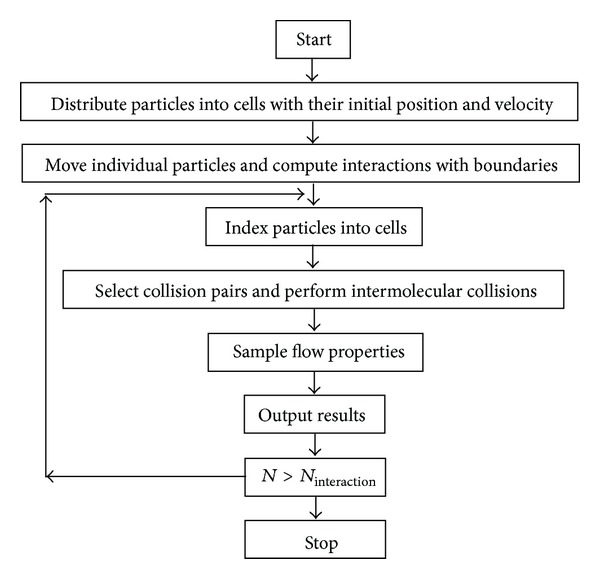
DSMC flowchart.

In DSMC, gas is represented by the velocity components and positions of a large number of simulated molecules. The position coordinates, velocity components, and internal states of the simulated molecule data are stored. These parameters are updated with time because the molecules are concurrently followed by representative collisions and boundary interactions in the simulated domain [12]. The primary approximation of DSMC is that molecular motions and intermolecular collisions are decoupled over short time intervals. The DSMC's flowchart in the current study is shown in Figure 1.

The core of the DSMC algorithm consists of four primary processes: moving the particles, indexing and cross-referencing the particles, simulating collisions, and sampling the flow field [9].

### 2.1. Moving the Particles

In the first process, all the simulated molecules are moved through distances, which are proportionate to their velocity components and discrete time step. Appropriate action is taken if the molecule crosses boundaries, such as solid surfaces and symmetry boundary. Collisions with surfaces can be treated as being fully secular, fully diffuse, or any combination of the two. Secular collisions involve a simple reversal of the molecular velocity component normal to the incident surface. Diffused collisions cause a random reorientation of the reflected molecule, where the postcollision velocity is based on surface temperature.

### 2.2. Indexing and Tracking the Particles

The second DSMC process involves indexing and tracking the particles. This molecular referencing scheme is needed as prerequisite for the next two steps: modeling the collisions and sampling the flow field.

### 2.3. Simulation of Collisions

The third step, simulating collisions, is an important process. And it is different from the MD (molecular dynamics) method. In DSMC simulation, elastic collision model is used in which no interchange of translational and internal energy is considered. And also, in the collision, linear momentum and energy are conserved.

#### 2.3.1. Impact Parameters and Collision Cross Section

In homogenous gas, the probability of two molecules collision is proportional to their relative speed and total collision cross section. In DSMC, cross section *σ* is defined as [13]

(3) $$\begin{array}{c} \sigma =\frac{b}{\sin\chi }\left|\frac{db}{d\chi }\right|. \end{array}$$

**Figure 2:**
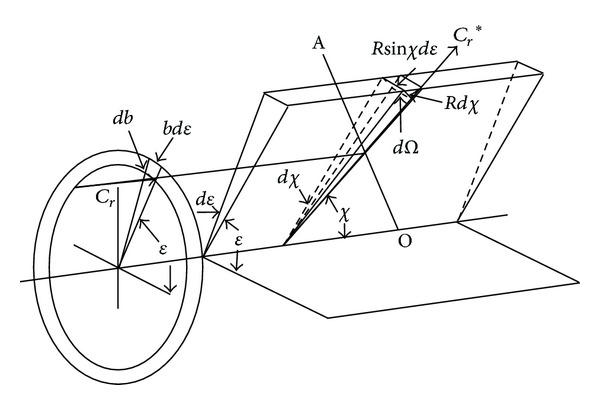
Illustration of the impact parameters [4].

Here, *b* is the distance of the closest approach to the undisturbed trajectories and *χ* is the angle of deflection, as shown in Figure 2.

Then, the total collision cross section can be written as

(4) $$\begin{array}{c} \sigma_{T}=2\pi \int_{0}^{\pi }\sigma \sin\chi d\chi =2\pi \int bdb. \end{array}$$

Here, the distance of the closest approach *b* is given in different molecular model. In the current paper, three molecular models, including HS, VHS, and VSS, are used in the simulation.

*(1) HS Model*. In the HS model, the intermolecular force is effective at

(5) $$\begin{array}{c} r=\frac{1}{2}\left(d_{1}+d_{2}\right)=d_{12}. \end{array}$$

**Figure 3:**
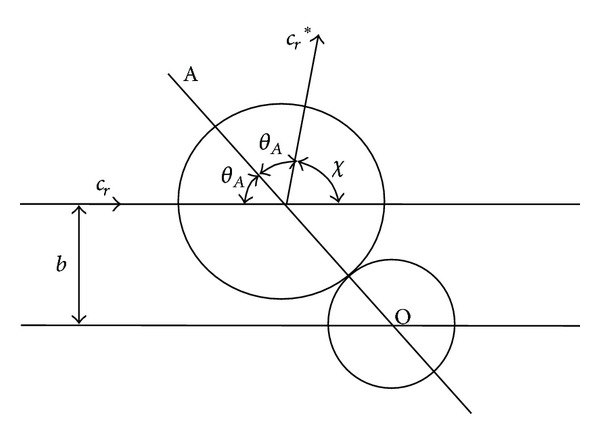
Collision geometry of HS molecules [4].

Then, as Shown in Figure 3, we can get the closest approach *b*

(6) $$\begin{array}{c} b=d_{12}\cos\left(\frac{1}{2}\chi \right),\\ \left|\frac{db}{d\chi }\right|=\frac{1}{2}d_{12}\sin\left(\frac{1}{2}\chi \right),\\ \sigma =\frac{d_{12}^{2}}{4}. \end{array}$$

Therefore, the total collision cross section is defined as

(7) $$\begin{array}{c} \sigma_{T}=\pi d_{12}^{2}. \end{array}$$

In HS model, the molecule diameter can be calculated as

(8) $$\begin{array}{c} d={\left[\frac{\left(5/16\right){\left(mkT_{\text{ref}}/\pi \right)}^{1/2}}{\mu_{\text{ref}}}\right]}^{1/2}, \end{array}$$

where  *μ*_ref_ is the reference viscosity at temperature *T*_ref_ and *m* is the molecular mass.

*k* is the Boltzmann constant and *k* = 1.380658 × 10^−23^ J/K.

*(2) VHS Model*. In the VHS model, molecular diameter *d* is defined as a function of relative velocity *c*_*r*_; that is,

(9) $$\begin{array}{c} d_{\text{VHS}}=d_{\text{ref}}{\left(\frac{c_{r,\text{ref}}}{c_{r}}\right)}^{\xi }={\left[\frac{\left(15/8\right){\left(m/\pi \right)}^{1/2}{\left(kT_{\text{ref}}\right)}^{\omega }}{\Gamma \left(\left(9/2\right)-\omega \right)\mu_{\text{ref}\;}\epsilon_{t}^{\omega -(1/2)}}\right]}^{1/2}. \end{array}$$

Here, *μ*_ref_ is the reference viscosity at temperature *T*_ref_. *ω* is the power law, which is expressed as *ω* = (1/2) + *ξ*. *ξ* is relative speed exponent of  VHS model and it can be adjusted to meet the power law *μ* ∝ *T* ^*ω*^. *ϵ*_*t*_ is the relative mean kinetic energy expressed as *ϵ*_*t*_ ≡ (1/2)*m*_*r*_ *c*_*r*_^2^. *c*_*r*_ is relative speed between the collision molecules. *c*_*r*,ref_ is reference relative speed between the collision molecules.

In the VHS model, the closest approach *b* can be defined as

(10) $$\begin{array}{c} b=d_{\text{VHS}}\cos\left(\frac{1}{2}\chi \right). \end{array}$$

The total collision cross section is expressed as

(11) $$\begin{array}{c} \sigma_{T}=\sigma_{T,\text{ref}}{\left(\frac{d_{\text{VHS}}}{d_{\text{ref}}}\right)}^{2}, \end{array}$$

where *σ*_*T*,ref_ is reference collision cross section at the reference molecule diameter *d*_ref_. 

*(3) VSS Model.* In the VSS model, molecular diameter *d* is defined as

(12) $$\begin{array}{c} d_{\text{VSS}}={\left[\frac{5\left(\alpha +1\right)(\alpha +2){\left(m/\pi \right)}^{1/2}{\left(kT_{\text{ref}}\right)}^{\omega }}{16\alpha \Gamma \left((9/2)-\omega \right)\mu_{\text{ref}\;}\epsilon_{t}^{\omega -(1/2)}}\right]}^{1/2}. \end{array}$$

Here, *α* is the scattering coefficient. (In the case where *α* = 1, the VSS model is the same as the VHS model.)

In the VSS model, the closest approach *b* is defined as

(13) $$\begin{array}{c} b=d_{\text{VSS}}\cos^{\alpha }\left(\frac{1}{2}\chi \right). \end{array}$$

The total collision cross section is defined as

(14) $$\begin{array}{c} \sigma_{T}=\pi d_{\text{VSS}}^{2}. \end{array}$$

#### 2.3.2. Gas-Surface Interaction

 Diffuse reflection [14] with full accommodation to the surface temperature is implemented in the simulation. It means that molecules diffused under the Maxwell distribution condition at *T* = *T*_*W*_ during the simulation.

### 2.4. Sampling

The sampling of the macroscopic flow properties is the final process in DSMC. The spatial coordinates and velocity components of the molecules in a particular cell are used to calculate the macroscopic quantities at the geometric center of the cell. Consider

(15) $$\begin{array}{c} \rho =n\;m,\\ P=\rho \bar{{\left(c-\bar{c}\right)}^{2}}, \end{array}$$

where *n* is the number density of the molecules, *c* is the velocity of the molecule, and $\bar{c}$ is the average velocity of the molecule.

### 2.5. Parameters

The parameters selected in the present paper are pressure coefficient and the Stanton number:

(16) $$\begin{array}{c} C_{P}=\frac{P-P_{\infty }}{\left(1/2\right)\rho V_{\infty }^{2}},\\ S_{t}=\frac{q}{\left(1/2\right)\rho V_{\infty }^{3}}, \end{array}$$

where *P* is the flow-field pressure, *P*_*∞*_ is the far-field pressure of the flow field, *ρ* is the density of flow-field density, *V*_*∞*_ is the far-field velocity of the flow field, and *q* is the thermal flux of the solid surface.

## 3. Hypersonic Double-Cone Flow Simulation

### 3.1. Moss and Boyd's DSMC Predictions for Hypersonic 25°/55° Double-Cone Flow

**Figure 4:**
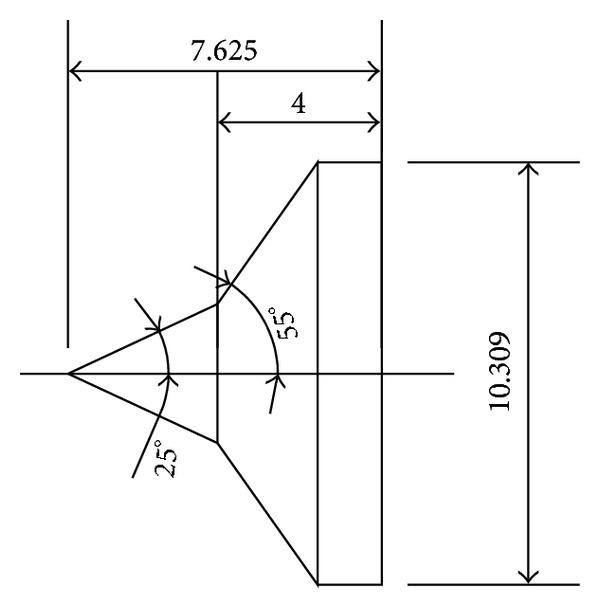
Number 1 configuration (in) (sharp nose 25°/55° double cone).

**Figure 5:**
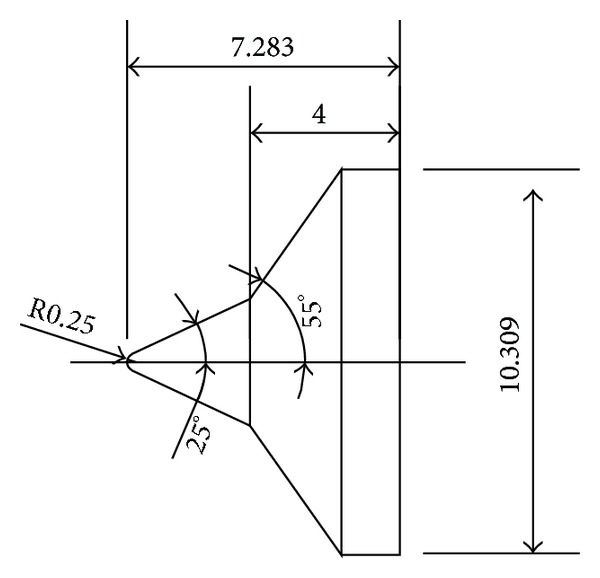
Number 2 configuration (in) (R0.25 blunt nose 25°/55° double cone).

**Figure 6:**
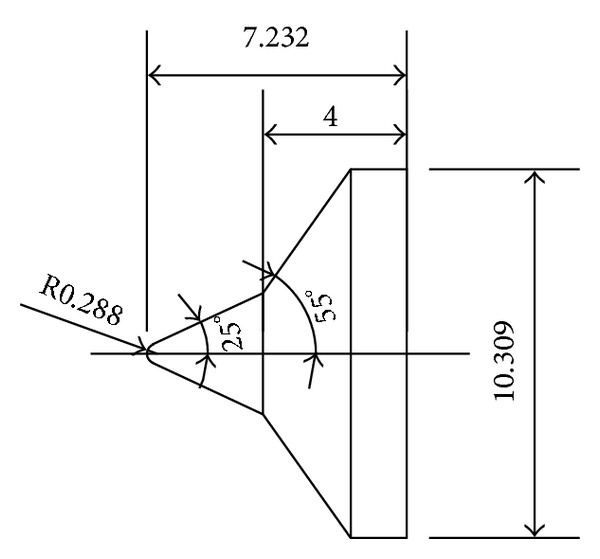
Number 3 configuration (in) (R0.288 blunt nose 25°/55° double cone).

**Figure 7:**
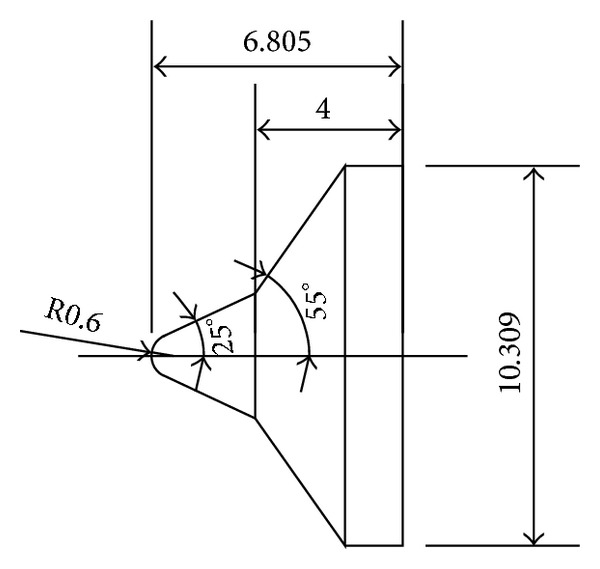
Number 4 configuration (in) (R0.6 blunt nose 25°/55° double cone).

**Figure 8:**
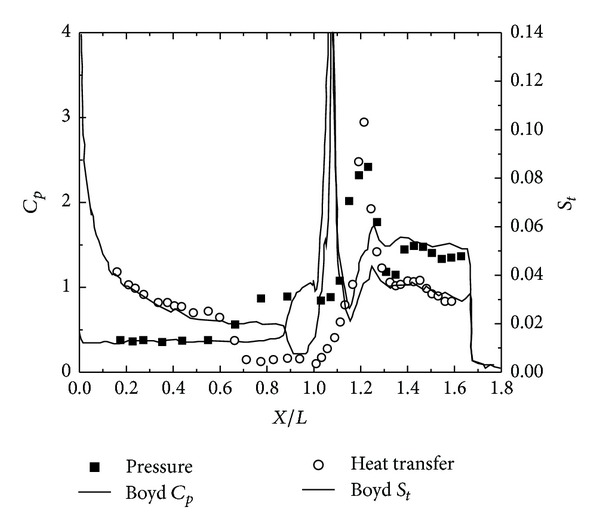
Boyd's DSMC for Run number 35 [1, 15].

To validate the DSMC method, three 25°/55° hypersonic double-cone configurations are selected under five conditions. The experimental data, with four configurations as shown in Figures 4, 5, 6, and 7 and, are reported in [1] in which Moss and Boyd's DSMC predictions are compared with the experimental data under some experimental conditions. Boyd's predictions using the DSMC code for Run number 35 are shown in Figure 8. These predictions clearly underestimated the length of the separated region, and although the heating in the forebody is slightly underpredicted, a good agreement existed between theory and experiment for the pressures and the heat transfer downstream of the shock/shock interaction in the second cone [12].

**Figure 9:**
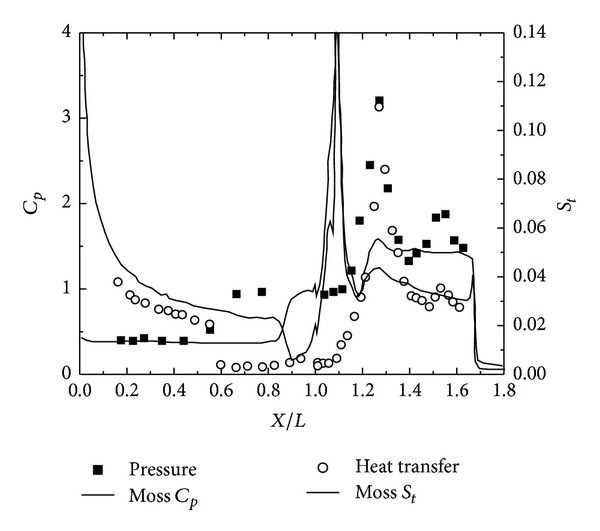
Moss's DSMC for Run number 28 [8].

**Figure 10:**
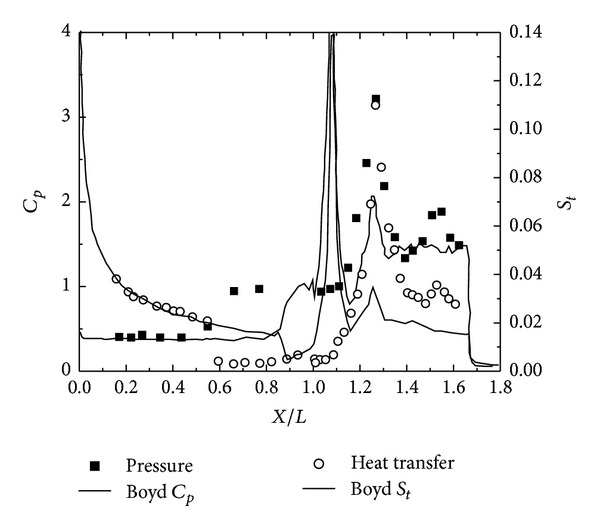
Boyd's DSMC for Run number 28 [15].

Figures 9 and 10 show the results obtained using the DSMC method by Moss [8] and Boyd [15], which significantly underpredicted the scale of the interaction region and the position and magnitude of the properties in and downstream of the region of shock/shock interaction. Moss calculations, shown in Figure 9, indicated that the predicted separated region is approximately 25% of the length measured in the experiment. Downstream of the interaction regions, the pressure and the heating levels were relatively well predicted. Boyd's solution for Run number 28 also significantly underpredicted the length of the separated region, and unlike Moss's calculation, the heat transfer rates both ahead and downstream of the interaction were underpredicted. Significant improvement of these calculations is doubtful even with the incorporation of vibrational nonequilibrium effects.

### 3.2. Our DSMC Simulation

In [1], Run numbers 26, 32, 33, 36, and 38 were not simulated by DSMC and CFD. Therefore, we selected these cases to validate the DSMC code with the HS, VHS, and VSS models.

The computational grid, employed for Run numbers 26, 32, 33, 36, and 38, consists of 2,048 cells along the body by 512 cells normal to the body. This grid is the same as that of Boyd's DSMC simulation [15]. The base time step for the simulation was 10^−9^ s, although this was reduced in very high density regions. The solutions were obtained using approximately 6.4 million particles and averaged over a sampling interval of 0.5 ms. At this time, the solution had attained steady state. In particular, the size of the separation region did not change.

The properties of nitrogen molecule, used in the current study, are presented in Table 2.

#### 3.2.1. DSMC Simulation of Run Numbers 26 and 36

In this section, the DSMC simulation result of Run numbers 26 and 36 is presented. As shown in Table 3, these two cases were conducted in the same Ma and Re conditions with sharp nose and R0.25 in blunt nose, respectively.

**Figure 11:**
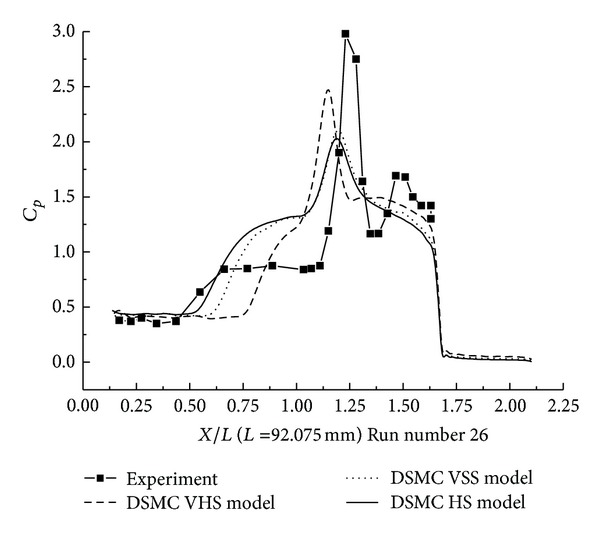
DSMC simulation of Run number 26 (*C*_*P*_).

**Figure 12:**
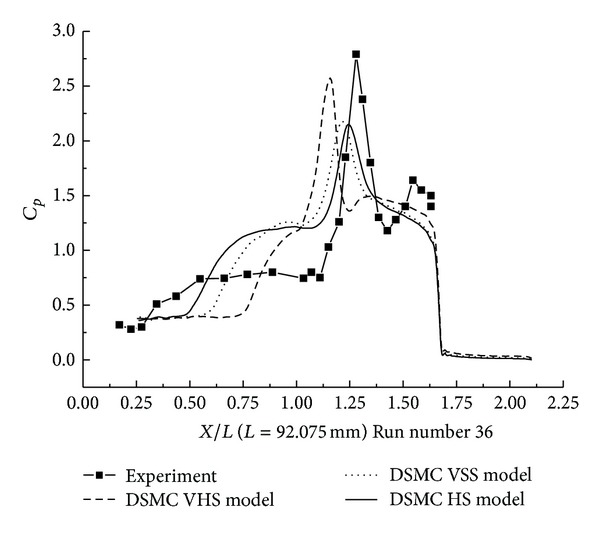
DSMC simulation of Run number 36 (*C*_*P*_).

**Figure 13:**
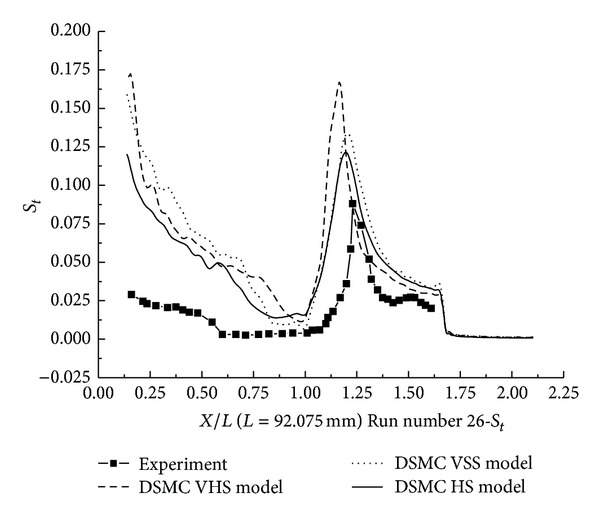
DSMC simulation of Run number 26 (*S*_*t*_).

**Figure 14:**
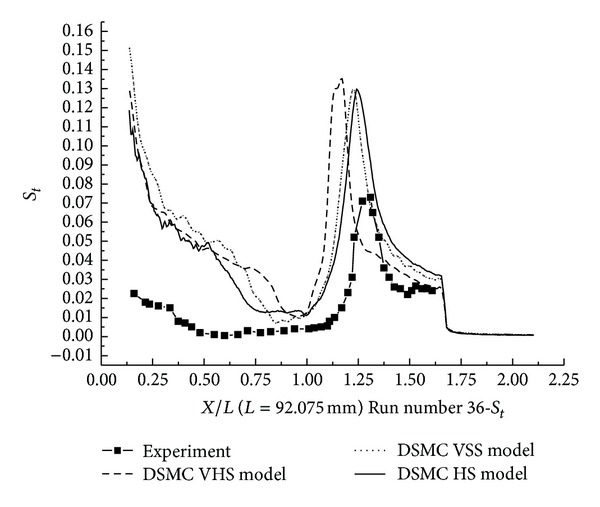
DSMC simulation of Run number 36 (*S*_*t*_).

The pressure coefficient and the Stanton number, obtained with HS, VHS, and VSS, are shown in Figures 11–14. These results underpredict the scale of interaction region and the position, and also the magnitude of the parameters is underpredicted in and downstream. The HS calculation shown in Figure 11 for Run number 26 indicates that the predicted interaction region is approximately 76% of the measured experimental length. Those of VHS and VSS are 34% and 62%, respectively. The HS results, shown in Figure 12 for Run number 36, indicate that the predicted interaction region is approximately 76% of the measured experimental length. Those of VHS and VSS are 30% and 57%, respectively. These predictions are much improved compared with Moss's DSMC calculation, which was only 25% of the length measured in the experiment [8]. The results of three models overpredicted the ahead pressure coefficient peak value. In the downstream of the interaction regions, the pressure levels got by the three models were relatively well predicted. And also, the three models overpredicted the Stanton number in comparison with the experimental data. The position of the Stanton number peak was well predicted by the three models.  Figure 13 shows that the peak values of the Stanton number using HS, VHS, and VSS are approximately 142%, 185.7%, and 148.5%, respectively, of the data measured in the experiment for Run number 26.  Figure 14 shows that the peak values of the Stanton number using HS, VHS, and VSS are approximately 178%, 192.8%, and 178%, respectively, of the data measured in the experiment for Run number 36.

#### 3.2.2. DSMC Simulation of Run Number 33

In this section, the DSMC simulation result of Run number 33 is presented. As shown in Table 4, Run numbers 33 and 36 were conducted in the same configuration with different Kn condation.

**Figure 15:**
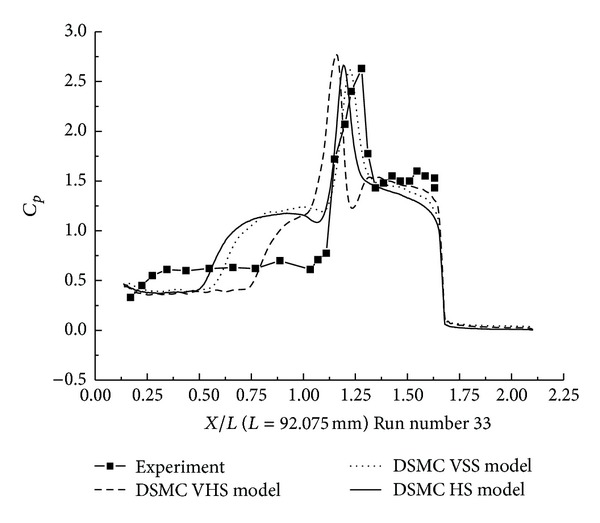
DSMC simulation of Run number 33 (*C*_*P*_).

**Figure 16:**
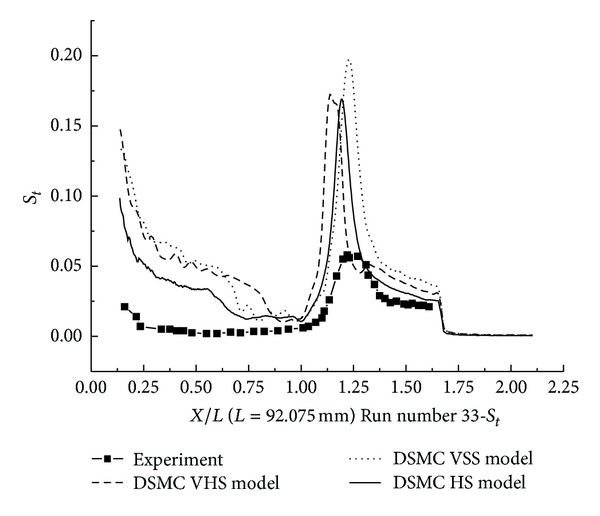
DSMC simulation of Run number 33 (*S*_*t*_).

The pressure coefficient and the Stanton number, obtained from HS, VHS, and VSS using the DSMC method, are shown in Figures 15 and 16. These results also underpredicted the scale of interaction region and position, magnitude of the properties in and downstream of the shock interaction region. The HS result, shown in Figure 15, indicates that the predicted interaction region is approximately 53.3% of the length measured in the experiment. Those of VHS and VSS are 26.6% and 46.6%, respectively. These predictions are not improved in comparison with Run numbers 26 and 36 calculations. The three models' calculation also overpredicted the ahead pressure coefficient peak value. In the downstream of the interaction region, the pressure levels from three models are relatively well predicted. Also, three models overpredicted the Stanton number compared with the experimental data. But, the position of the Stanton number peak is well predicted in the three models' calculation.  Figure 16 shows that the peak values of the Stanton number in HS, VHS, and VSS are approximately 290%, 309%, and 345%, respectively, of the data measured in the experiment for Run number 33.

#### 3.2.3. DSMC Simulation for Run Numbers 32 and 38

In this section, the DSMC simulation results for Run numbers 32 and 38, as shown in Table 5, are presented. Run numbers 33, 38, and 36 were conducted in the same configuration with R0.25 in blunt nose.

**Figure 17:**
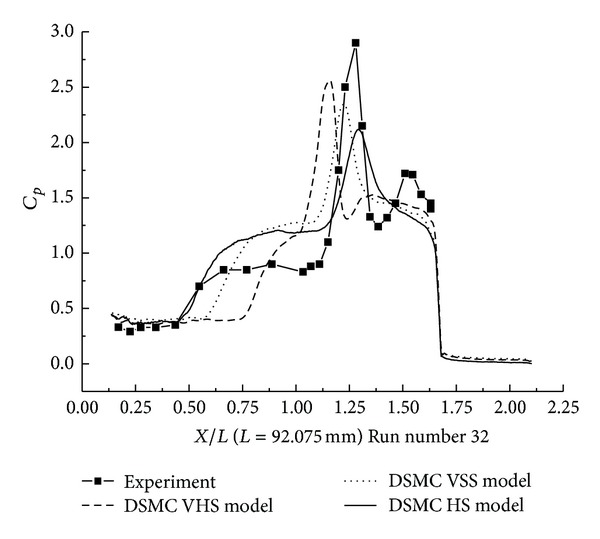
DSMC simulation of Run number 32 (*C*_*P*_).

**Figure 18:**
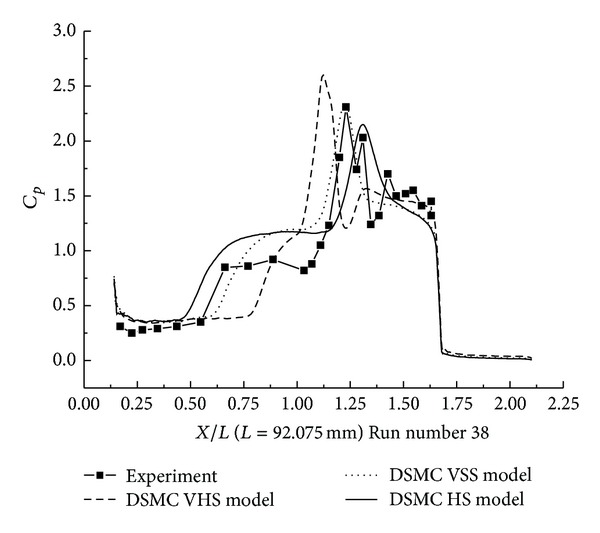
DSMC simulation of Run number 38 (*C*_*P*_).

**Figure 19:**
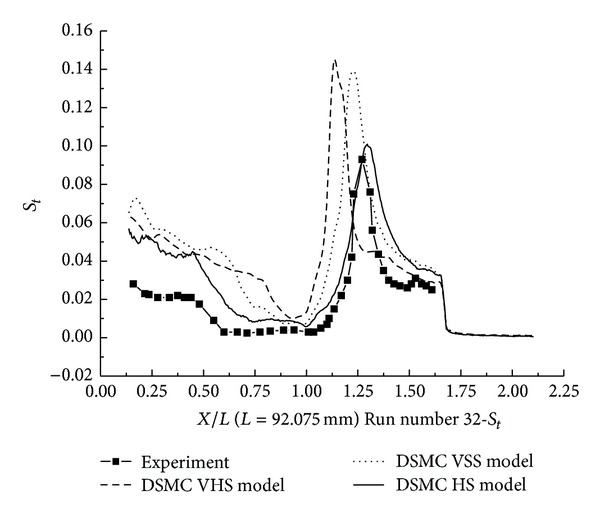
DSMC simulation of Run number 32 (*S*_*t*_).

**Figure 20:**
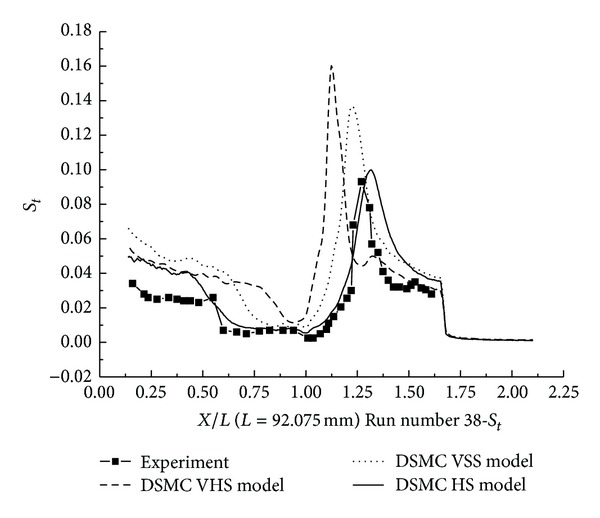
DSMC simulation of Run number 38 (*S*_*t*_).

The pressure coefficient and the Stanton number obtained by HS, VHS, and VSS using the DSMC method are shown in Figures 17–20. These results well predicted the scale of the interaction region and the position, magnitude of the properties in and downstream of the shock interaction region. The HS result, shown in Figure 17 for Run number 32, indicates that the predicted interaction region is approximately 100% of the length measured in the experiment. Those of VHS and VSS are 41% and 69%, respectively. The HS result, shown in Figure 18 for Run number 38, indicates that the predicted interaction region is approximately 106.8% of the length measured in the experiment. Those of VHS and VSS are 27.6% and 68.96%, respectively. These predictions are improved in comparison with Run numbers 26 and 36 calculations. Also, the three models' calculation overpredicted the ahead pressure coefficient peak value. And in the downstream of interaction region, the pressure levels from the three models are relatively well predicted. The three models also overpredicted the Stanton number in comparison with the experimental data. And also, the position of the Stanton number peak is well predicted in the three models' calculation.  Figure 19 shows that the peak values of the Stanton number in HS, VHS, and VSS are approximately 105.2%, 157.8%, and 147.4%, respectively, of the data measured in the experiment for Run number 32.  Figure 20 shows that the peak value of the Stanton number in HS, VHS, and VSS are approximately 105.2%, 168.4%, and 142.1%, respectively, of the data measured in the experiment for Run number 38.

Table 6 shows that the DSMC error depends on the Kn number and the models employed for intermolecular interaction. With the increase in the Kn number, the DSMC error decreases. The error is the smallest in the HS model compared with those in the VHS and VSS models. With the Kn number increasing, the Stanton number DSMC error decreases. When the Kn number is in the level of 10^−4^, the DSMC error for pressure coefficient and Stanton number is controlled within 10%.

## 4. Type IV Shock Interaction Simulation

**Figure 21:**
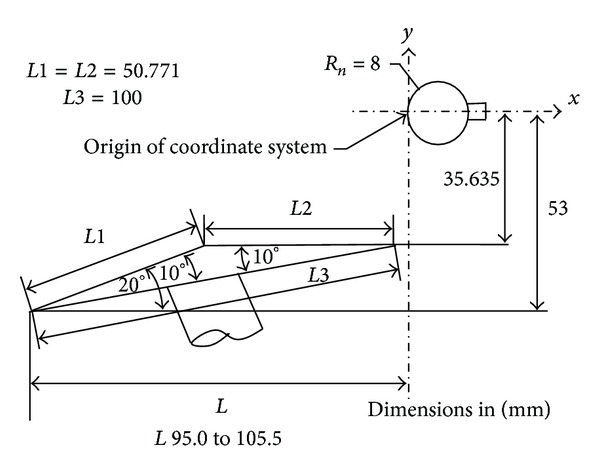
Configuration of Edney's Type IV shock interaction.

In the present study, results of the numerical simulations for Mach 10 air flow, as shown in Table 7, were presented for a range of Edney's type IV shock interaction. The experimental data for validation were obtained from [16]. The configuration is shown in Figure 21.

The results of the grid sensitivity investigation are presented in [16]. The number of cells/subcells used for our simulation was 97,060/958,490, proven in [16] to be the finest. The computational time step was 1.2 ns. The simulation consisted of 1.56 × 10^5^ time steps to ensure steady state conditions.

**Figure 22:**
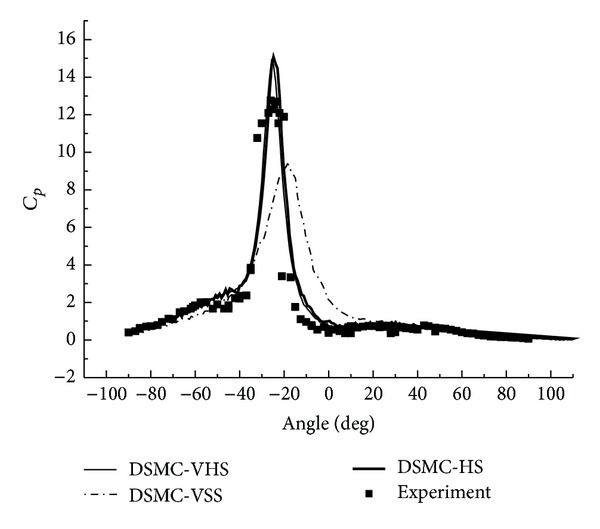
DSMC of type IV shock interaction (*C*_*P*_).

**Figure 23:**
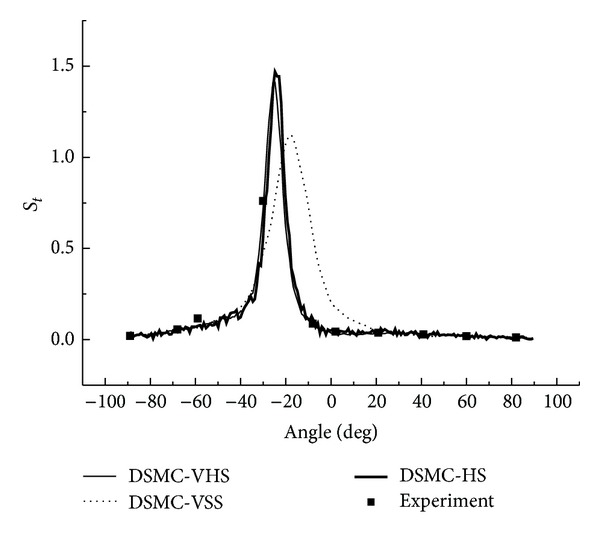
DSMC of type IV shock interaction (*S*_*t*_).

The DSMC data of the pressure coefficient and the Stanton number are shown in Figures 22 and 23. The agreement between the DSMC calculation and the experiment is generally good. Both HS and VHS models gain improvements in the errors. The error between the HS-DSMC data and the experimental data is controlled within 5%.

The result from the DSMC calculation for Edney's type IV shock interaction also confirmed the same conclusion. The error is the smallest in the HS model compared with those in the VHS and VSS models. In the region of large Kn number, DSMC using the HS model could be used in pressure and thermal prediction of hypersonic complex flow.

## 5. Conclusion

In the present study, the double-cone and Edney's type IV shock interactions were studied. Molecular models were employed with DSMC including the HS, VHS, and VSS models. Comparisons between DSMC and the experiment were also conducted.

Drawing firm conclusions from this study prior to full comparisons of these results is somewhat difficult. However, some issues are immediately clear from our attempt to compute these flows.

1. The DSMC method appears to predict qualitatively the structure of flow separation found in previous CFD calculations.
2. Investigation of the double-cone hypersonic flow reveals that the three collisions models can predict the pressure coefficient and the Stanton number trends. The agreement between the DSMC simulation and the experiment is the best in the HS collisions model.
3. Investigation of type IV shock interaction shows that the agreement between the DSMC simulation and the experiment is generally good in the HS and VHS collision models. However, it fails in the VSS model.
4. Both double-cone flow and type IV shock interaction simulations show that the DSMC error depends on the Kn number and the models employed for intermolecular interaction. Increasing the Kn number decreases the DSMC error. The error is the smallest for the HS model compared with those for the VHS and VSS models. When the Kn number is in the level of 10^−4^, the DSMC error for pressure coefficient, the Stanton number, and scale of interaction region is controlled within 10%.

## Figures and Tables

**Table 1:** Test case in [1].

Run	V_∞_(m/s)	ρ_∞_ (Kg/m^3^ ×10^3^)	P_∞_ (Pa)	Ma	Re/m (×10^5^)	Nose
26	2581	1.093	48.7	10.34	1.704	Sharp
28	2520	0.727	30.0	10.50	1.106	Sharp
35	2575	0.608	18.5	12.49	0.945	Sharp
32	2572	0.758	32.1	10.56	1.177	R0.250
33	2636	1.288	59.8	10.35	2.050	R0.250
36	2584	1.108	49.6	10.34	1.729	R0.250
38	2453	0.498	13.9	12.47	0.738	R0.288

**Table 2:** Properties of nitrogen molecule in DSMC.

Degree of freedom	5
Molecular mass × 10^27^ Kg	46.5
Viscosity coefficient	1.656
Viscosity index	0.74
*α* (for VSS model)	1.36
Molecular diameter (VHS) × 10^10^ m	4.17
Molecular diameter (VSS) × 10^10^ m	4.11
Molecular diameter (HS) × 10^10^ m	3.784

**Table 3:** Flow conditions for Run Numbers 26 and 36.

	Ma	Re/m	Kn
Run 26	10.34	1.704 × 10^5^	9.05 × 10^−5^
Run 36	10.34	1.73 × 10^5^	8.91 × 10^−5^

**Table 4:** Flow conditions for Run Numbers 33 and Numbers 36.

	Ma	Re/m	Kn
Run 33	10.35	2.05 × 10^5^	7.53 × 10^−5^
Run 36	10.34	1.73 × 10^5^	8.91 × 10^−5^

**Table 5:** Flow conditions for Run Numbers 32 and 38.

	Ma	Re/m	Kn
Run 32	10.56	1.177 × 10^5^	1.34 × 10^−4^
Run 38	12.47	0.738 × 10^5^	2.52 × 10^−4^

**Table 6:** Comparisons of calculations ((Value_DSMC_ − Value_experiment_)/(Value_experiment_)).

Run	Kn	The scale of interaction region (error in compared with measured data %)			Peak value of the Stanton number (error in compared with measured data %)		
		HS	VHS	VSS	HS	VHS	VSS
33	7.53 × 10^−5^	*46.7 *	73.4	53.4	*190 *	209	245
36	8.91 × 10^−5^	*24 *	70	43	*78 *	92.8	78
26	9.05 × 10^−5^	*24 *	66	38	*42 *	85.7	48.5
32	1.34 × 10^−4^	*0 *	59	31	**5.2**	57.8	47.4
38	2.52 × 10^−4^	*6.8 *	72.4	31.04	**5.2**	68.4	42.1

**Table 7:** Flow conditions of Edney's type IV shock interaction.

Ma	Re/m	Kn
10	1.03 × 10^5^	1.45 × 10^−4^

## References

[1] Holden MS, Wadhams TP, Harvey JK, Candler GV (2006). Comparisons between measurements in regions of laminar shock wave boundary layer interaction in hypersonic flows with navier-stokes and DSMC solutions.

[2] Olejniczak J, Candler GV Computation of hypersonic shock interaction flow fields.

[3] Candler GV, Nompelis I, Druguet M-C, Boyd ID, Wang W-L, Holden SM CFD validation for hypersonic flight: hypersonic double-cone flow simulations.

[4] Bird GA (1994). *Molecular Gas Dynamics and the Direct Simulation of Gas Flows*.

[5] Harvey, John K, Holden MS, Wadhams TP Code validation study of laminar shock/boundary layer and shock/shock interactions in hypersonic flow. Part B: comparison with navier-stokes and DSMC solutions.

[6] Gallis M, Roy C, Payne J, Bartel T DSMC and navier-stokes predictions for hypersonic laminar interacting flows.

[7] Candler GV, Nompelis I, Druguet M-C Navier-stokes predictions of hypersonic double-cone and cylinder-flare flow fields.

[8] Moss J DSMC computations for regions of shock/shock and shock/boundary layer interaction.

[9] Bird GA (2013). *The DSMC Method*.

[10] Woo M, Greber I (1999). Molecular dynamics simulation of piston-driven shock wave in hard sphere gas. *AIAA Journal*.

[11] Koura K, Matsumoto H (1992). Variable soft sphere molecular model for air species. *Physics of Fluids A*.

[12] Prasanth PS, Kakkassery JK (2006). Direct simulation Monte Carlo (DSMC): a numerical method for transition-regime flows: a review. *Journal of the Indian Institute of Science*.

[13] Tokumasu T, Matsumoto Y (1999). Dynamic molecular collision (DMC) model for rarefied gas flow simulations by the DSMC method. *Physics of Fluids*.

[14] Borisov SF, Capitelli M Progress in gas-surface interaction study.

[15] Boyd ID, Wang W-L Monte Carlo computations of hypersonic interacting flows.

[16] Moss JN, Pot T, Chanetz B, Lefebvre M DSMC simulation of shock/shock interactions: emphases on type IV interaction.

